# Potential of CBD Acting on Cannabinoid Receptors CB_1_ and CB_2_ in Ischemic Stroke

**DOI:** 10.3390/ijms25126708

**Published:** 2024-06-18

**Authors:** Iu Raïch, Jaume Lillo, Rafael Rivas-Santisteban, Joan Biel Rebassa, Toni Capó, Montserrat Santandreu, Erik Cubeles-Juberias, Irene Reyes-Resina, Gemma Navarro

**Affiliations:** 1 Department of Biochemistry and Physiology, Faculty of Pharmacy and Food Sciences, University of Barcelona, 08028 Barcelona, Spain; iraichpa7@ub.edu (I.R.); jrebaspa7@alumnes.ub.edu (J.B.R.); tonicapoquetglas@ub.edu (T.C.); 2001montse@gmail.com (M.S.); ecubelju7@alumnes.ub.edu (E.C.-J.); 2 Centro de Investigación en Red, Enfermedades Neurodegenerativas (CIBERNED), Instituto de Salud Carlos III, 28031 Madrid, Spain; jaumelillo@ub.edu (J.L.); rrivas@ub.edu (R.R.-S.); 3 Institute of Neuroscience, University of Barcelona (NeuroUB), Campus Mundet, Passeig de la Vall d’Hebron 171, 08035 Barcelona, Spain; 4 Department of Biochemistry and Molecular Biomedicine, School of Biology, University of Barcelona, 08028 Barcelona, Spain

**Keywords:** CB_1_R, CB_2_R, cannabinoids, hypoxia, ischemia

## Abstract

Stroke is one of the leading causes of death. It not only affects adult people but also many children. It is estimated that, every year, 15 million people suffer a stroke worldwide. Among them, 5 million people die, while 5 million people are left permanently disabled. In this sense, the research to find new treatments should be accompanied with new therapies to combat neuronal death and to avoid developing cognitive impairment and dementia. Phytocannabinoids are among the compounds that have been used by mankind for the longest period of history. Their beneficial effects such as pain regulation or neuroprotection are widely known and make them possible therapeutic agents with high potential. These compounds bind cannabinoid receptors CB_1_ and CB_2_. Unfortunately, the psychoactive side effect has displaced them in the vast majority of areas. Thus, progress in the research and development of new compounds that show efficiency as neuroprotectors without this psychoactive effect is essential. On the one hand, these compounds could selectively bind the CB_2_ receptor that does not show psychoactive effects and, in glia, has opened new avenues in this field of research, shedding new light on the use of cannabinoid receptors as therapeutic targets to combat neurodegenerative diseases such as Alzheimer’s, Parkinson’s disease, or stroke. On the other hand, a new possibility lies in the formation of heteromers containing cannabinoid receptors. Heteromers are new functional units that show new properties compared to the individual protomers. Thus, they represent a new possibility that may offer the beneficial effects of cannabinoids devoid of the unwanted psychoactive effect. Nowadays, the approval of a mixture of CBD (cannabidiol) and Δ^9^-THC (tetrahydrocannabinol) to treat the neuropathic pain and spasticity in multiple sclerosis or purified cannabidiol to combat pediatric epilepsy have opened new therapeutic possibilities in the field of cannabinoids and returned these compounds to the front line of research to treat pathologies as relevant as stroke.

## 1. Introduction

Stroke or cerebrovascular accident (CVA) appears when blood flow to a specific part of the brain drops, halts, or significantly impairs, potentially causing severe brain harm, even disability or death [1]. Stroke is mainly classified into two types: hemorrhagic infarction, which accounts for approximately 20% of all cases, in which there is a rupture of blood vessels, and ischemic infarction, in which there is an occlusion of blood vessels and it accounts for 80% of cases [2].

Molecular mechanisms by which damage occurs in a CVA are diverse, including decreased oxygen and nutrient supply to the affected and surrounding neuronal tissue [3], increased reactive oxygen species (ROS) [4], and augmented inflammation [5]. Consequently, there is an increase in apoptosis and neuronal death [6].

In fact, stroke is one of the leading worldwide causes of death, only surpassed by heart failure. It has been detected that the number of stroke cases increases dramatically after the age of 75 [7]. Currently, there is an important research effort to prevent this pathology and, on the other hand, to reduce the brain damage caused by stroke in order to avoid developing cognitive impairment, dementia, and other neurologic subsequent complications [8].

Existing anticoagulant treatments for prevention CVA have very dangerous side effects, including bleeding and exacerbation of hemorrhagic strokes. In fact, anticoagulants are not typically used as treatment for established ischemic stroke; instead, thrombolysis is primarily employed [9]. While there are established treatments available for acute ischemic stroke such as thrombolysis, these come with limitations and risks. In search of alternative therapies for stroke, the endocannabinoid system has emerged as a key target for therapeutic interventions. The endocannabinoid system consists of two cell-surface G-protein-coupled receptors (GPCRs), which are cannabinoid receptor type I (CB_1_R) and type II (CB_2_R), their endogenous ligands, known as endocannabinoids (mainly anandamide (AEA) and 2-arachidonoylglycerol (2-AG)), and the enzymes that control their biosynthesis and degradation [10]. The widespread distribution of cannabinoid receptors in the central nervous system (CNS), particularly the highly overexpressed CB_1_R in human stroke [11], paired with their anti-inflammatory and neuroprotective mediated signaling [12], presents a compelling case for exploring the promising benefits of cannabinoid compounds as a therapeutic strategy for stroke.

Currently, several applications of cannabinoid compounds are under investigation to prevent or decrease the harmful consequences of stroke. The study of Khaksar and Bigdeli found that cannabidiol significantly reduced infarct area and diminished proinflammatory factors in a rat model of transient focal cerebral ischemia [13]. Other treatments with cannabinoid compounds such as WIN55212-2, an agonist for CB_1_R and CB_2_R, or JWH-133, a selective agonist for CB_2_R, have been reported to reduce cerebral infarction volume both in adult and neonatal ischemia in hypoxia-ischemia animal models [14]. Overall, the literature suggest that cannabinoids exhibit neuroprotective effects in animal models of stroke and may represent a promising therapeutic option for stroke treatment.

Additionally, the protective responses of microglia after CVA, such as debris clearance at early stages and anti-inflammatory activity at later stages, are important factors to be considered [15]. Expression of CB_1_ and, especially, CB_2_ receptors has been detected in microglial cells [16]. Furthermore, it has been determined that CB_1_R and CB_2_R are downregulated in the proinflammatory phenotype of microglia (M1) while they are overexpressed in the anti-inflammatory microglia phenotype (M2) [17].

Unfortunately, not all findings in cannabinoid research are positive. Some publications suggest a link between the increased prevalence of stroke in young people and cannabis abuse [18]. In fact, cannabis use as a recreational drug has been linked to an increased risk of stroke [19]. The underlying mechanisms by which cannabinoids contribute to stroke involve an increased likelihood of ischemic infarction, primarily due to the enhanced platelet aggregation that promotes thrombus formation. Although it is important to note that there are cannabinoid compounds that favor platelet aggregation such as anandamide or 2-AG, others such as CBD or WIN-55,212-2 do not seem to accelerate coagulation [20]. Additionally, cannabis use has been shown to increase the risk of hemorrhagic infarction, likely due to the drug’s ability to elevate heart rate and blood pressure. However, some cannabinoid compounds, such as CBD, not only do not increase blood pressure but, under certain conditions, they are able to lower it [21]. Furthermore, it is also necessary to consider the vasoconstrictor power of some cannabinoid compounds that can also promote the development of stroke [22]. Nonetheless, the considerable potential of cannabinoid compounds to improve the aftermath of stroke should not be disregarded.

## 2. Functional Role of CB_1_R in Stroke

It has been reported that, after ischemia, there is an increase in the concentration of anandamide (AEA) and other endocannabinoids in brain tissue [23,24]. In other words, cannabinoid signaling is altered.

The CB_1_R is the most expressed receptor in the central nervous system [25]. Its involvement in physiological and pathological events justifies its central role as a possible therapeutic key in many diseases. Unfortunately, the psychoactive side effects generated by activation of CB_1_R in the brain have limited the use of orthosteric CB_1_R ligands as drugs [26]. In addition to the main binding site, the CB_1_R also has a modulatory binding pocket in the allosteric site. In Yang et al.’s study, information is provided about structural dynamics and energetics underlying CB_1_R activation and allosteric modulation [27]. To address the limitations of orthosteric ligands, the use of allosteric cannabinoid ligands represents a promising alternative. Allosteric modulation of the CB_1_R provides novel opportunities for therapeutic interventions.

This receptor is abundantly expressed in the axons and presynaptic terminals of neurons within the amygdala, hippocampus, cortex, basal ganglia output pathways, and cerebellum [28,29,30]. CB_1_R expression is altered both in patients and animal models of stroke [31,32,33]. Different investigations have described an increase in CB_1_R expression after an ischemic episode [11,32]. A study conducted on patient samples demonstrated an increased immunohistochemical labeling of CB_1_R in the ischemic region [11] and another study observed that administering a calorie-restricted diet to mice resulted in increased expression of CB_1_R in the striatum and hypothalamus and conferred protection against ischemia [34]. Conversely, 5 h of permanent middle cerebral artery (MCA) occlusion did not affect the density of CB_1_R binding sites in male rats [35]. Although, in gerbils exposed to a short period of global ischemia (2.5 min), a decrease in the presence of CB_1_R in the CA1 and CA3 regions of the hippocampus has been described [34].

**Figure 1:**
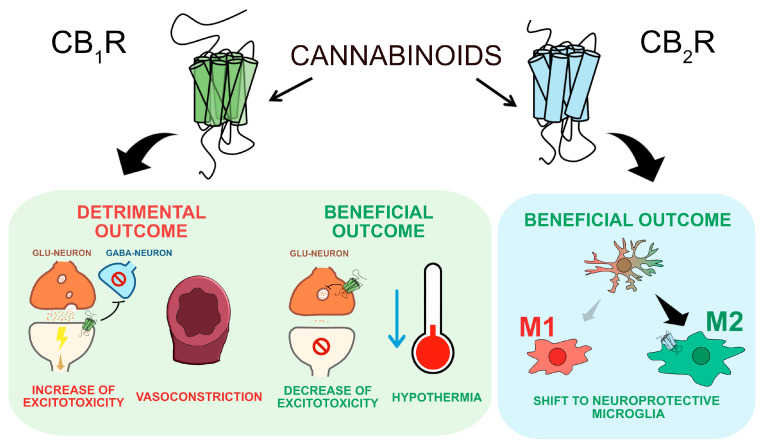
Beneficial and detrimental effects of cannabinoid CB_1_ and CB_2_ receptor activation in the context of ischemic stroke.

The administration of pharmacological treatments targeting CB_1_R modulation yields controversial results. Several studies have demonstrated that CB_1_R antagonism exerts neuroprotective effects in animal models of stroke [32,36]. In a rat model of global brain ischemia, treatment with AM251, a CB_1_R antagonist, exhibits neuroprotective effects in damaged regions by reducing neuronal death and enhancing performance in behavioral tests [37]. There are possible mechanisms that may explain why reduced CB_1_R activation causes a decrease in ischemic injury. CB_1_R are present on the terminals of GABAergic interneurons in the hippocampus. Activation of this receptor leads to decreased inhibitory neurotransmission, potentially exacerbating excitotoxicity. Consequently, CB_1_R blockade would mitigate this excitotoxicity, thereby providing neuroprotection (Figure 1).

On the other hand, the absence of CB_1_R resulted in a heightened severity of ischemia, indicating the involvement of CB_1_R-mediated regulation of cerebral vessels in exerting protective effects [38]. Additionally, administration of the selective CB_1_R agonist ACEA, following both intracerebral and intraperitoneal routes (at doses of 10 μM and 1 mg/kg, respectively), has demonstrated neuroprotective effects in the endothelin-induced embolic middle cerebral artery occlusion (eMCAO) and permanent middle cerebral artery occlusion (pMCAO) models, resulting in reduced neuronal death and brain injury volume [31,32]. The mechanisms underlying the protective effects of CB_1_R activation may be associated with the ability of CB_1_R activation to confer protection against glutamate-induced excitotoxicity (Figure 1). This hypothesis is supported by the effects of CB_1_R agonist in cell culture models. For example, presynaptic CB_1_R activation hyperpolarized the neuronal membrane, causing an inhibition of the voltage-operated calcium channels and an inhibition of glutamate release [38].

In a study, it was observed that CB_1_R activation contributes to a reduction in glutamatergic signaling subsequent to oxygen and glucose deprivation (OGD) in hippocampal slices [39].

In vivo studies corroborate in vitro findings, showing that activation of the cannabinoid receptor provides protection in models of excitotoxic injury. Δ^9^-THC diminishes neuronal damage in neonatal rats injected with a Na^+^-K^+^ ATPase inhibitor, which induces secondary excitotoxicity. These effects of Δ^9^-THC were prevented by co-administration of the CB_1_R antagonist rimonabant [40] (Figure 1). Furthermore, administration of the general cannabinoid agonist CP55940 facilitated the association of the tight junction protein zonula occludens-1 (ZO-1) with CB_1_R via the NH_2_-terminus of ZO-1. Activation of CB_1_R restored the expression of ZO-1 and preserved the integrity of the blood–brain barrier by promoting the reformation of continuous, uniform, linear tight junction structures [41].

Activation of the CB_1_R receptor elicits hypothermic responses [40], which have demonstrated neuroprotective effects across various ischemic models [42] (Figure 1). Furthermore, activation of the CB_1_R diminishes edema. Edema formation in the brain is frequently observed during ischemia, reperfusion, and other types of brain injury [43]. Multiple potential mechanisms underlie the capacity of CB_1_R agonists to mitigate edema, encompassing lowered systemic blood pressure and heightened release of glucocorticoids in reaction to stress [44,45].

## 3. Functional Role of CB_2_R in Stroke

The cannabinoid receptor type 2 is a seven transmembrane G-protein-coupled receptor (GPCR) and one of the known cannabinoid receptors found in the human body [46] that plays a key role in regulating various physiological processes, including pain, appetite, and mood [47].

Cannabinoid receptors are the target for exogenous and endogenous cannabinoids such as AEA and 2-araquidonilglicerol (2-AG). When these ligands interact with the CB_2_ receptor, they can modulate its function, leading to a range of potential therapeutic benefits [48]. For instance, cannabidiol acts as a negative allosteric modulator of CB_2_R, inducing conformational changes in such a way that biases the effect of orthosteric agonists [49].

Originally, it was thought that the CB_2_R was only expressed in peripheral tissue immune cells [50]. However, recent studies have shown CB_2_R expression within neurons, specifically in dopaminergic neurons of the ventral tegmental area, hippocampal glutamatergic neurons, and brain stem neurons. CB_2_R expression has also been reported in other cell types of the central nervous system, such as activated microglia [51,52]. Evidence suggests that targeting CB_2_ receptors can reduce inflammation, decrease spasticity, and inhibit neuronal apoptosis [53,54]. In the case of stroke, the CB_2_ receptor is also associated with positive outcomes.

Recent studies have focused on exploring the potential therapeutic effects of targeting cannabinoid receptor type II (CB_2_R) in individuals suffering from stroke. In an animal model, CB_2_R agonism was neuroprotective and increased neural progenitor cell migration in vitro [55]. Moreover, a pretreatment with CB_2_R agonists was able to suppress neurodegeneration in a rat model with ischemic stroke [56].

While research into the potential use of CB_2_R activation in the management of stroke is still in its early stages, the findings to date are promising. CB_2_R activation has been shown to have a range of neuroprotective benefits in a stroke context, including anti-inflammatory and antioxidant effects, which may be favorable in cases of stroke [55]. Inflammation is a key contributor to the progression of brain damage following a stroke, and reducing inflammation may help to limit the extent of this damage [57].

CB_2_ receptor activation has been shown to reduce the production of proinflammatory cytokines that contribute to the inflammatory response [58]. This is due to the high CB_2_ receptor expression in microglial cells, which are immune cells in the brain that play a key role in the response to injury and polarization [59]. The stimulation of CB_2_R activity attenuates proinflammatory M1 macrophage polarization, increasing the anti-inflammatory M2 markers (Figure 1); thus, CB_2_ receptor contributes to reducing edema development, enhances cerebral blood flow, and improves neurobehavioral outcomes [60]. Additionally, it has been observed that the activation of CB_2_R induces a reduction in glutamate-mediated excitotoxicity, which can impair the function of neurons and cause further damage to the brain [61].

Further research is needed to fully understand the potential of CB_2_ activation as a treatment option for stroke, but these initial findings suggest that it may have a role to play in the management of this condition.

## 4. Implication of CB_2_R–5HT_1A_R Complexes in Stroke

CB_2_ and 5HT_1A_ receptors have been shown to interact, forming macromolecular complexes, namely CB_2_R–5HT_1A_R-Het [62]. CB_2_R–5HT_1A_R-Het expression is highly controlled at different stages of brain development. At birth, a relatively high number of these structures are present but, as the nervous system develops, their presence rapidly decreases [63].

CBD is a phytocannabinoid that interacts with several receptors, among them the cannabinoid receptors CB_1_ and CB_2_ [64]. At micromolar concentrations, CBD can bind to the orthosteric site of CB_2_R, acting as a low-potency agonist and, at nanomolar concentrations, it can interact with the non-orthosteric sites, acting as an allosteric modulator [65,66]. Besides the cannabinoid receptors, it is known that CBD activates serotonin 5HT_1A_ receptors [67]. CBD has long been considered as a neuroprotective molecule. There is an increasing number of studies showing that the neuroprotective power of CBD also plays an important role in stroke pathology. An analysis of more than 34 preclinical studies examining the effect of CBD after an episode of stroke concluded that CBD significantly reduced infarct size and improved functional recovery, producing its effects through both CB_1_R and CB_2_R and also through the serotonin receptor 5HT_1A_R [68,69].

It has been observed that many of the effects caused by the phytocannabinoid are attributed to the activation of the serotonergic pathway. In their research, Kosari-Nasab et al. sought to identify the regulating role of 5-HT1AR in depression-related behaviors after mild traumatic brain injury (mTBI) in mice. Stimulation of 5-HT1AR with a subthreshold dose of the agonist 8-OH-DPAT caused a notable reduction in depression-like behaviors, whereas blocking the 5-HT1A receptor with a subthreshold dose of the antagonist WAY-100635 led to a significant rise in depression-like symptoms in mice subjected to mTBI [70].

In a study aiming to investigate whether CBD had any effect on the formation of heteromeric complexes between CB_2_ and 5HT_1A_ receptors, a bioluminescence resonance energy transfer (BRET) assay was performed in the absence and in the presence of 200 nM CBD, and cannabigerol (CBG) was used as a reference compound. Notably, pretreatment with 200 nM CBD significantly increased the maximum BRET signal (BRETmax) and apparent affinity (BRET_50_) [62]. This suggests that CBD either increases the number of formed complexes or causes a structural rearrangement in the CB_2_R–5HT_1A_R receptor complex. In contrast, pretreatment with 200 nM of CBG only increased the BRETmax without significantly affecting the BRET_50_.

Authors then examined the expression levels of the heteromer and the impact of heteromer formation on receptor functionality. First, β-arrestin 2 recruitment was analyzed by BRET in HEK-293T cells expressing either CB_2_R-YFP, 5HT_1A_R-YFP, or CB_2_R-YFP and 5HT_1A_R together, along with β-arrestin 2-RLuc. The experiments conducted on CB_2_R-expressing cells indicated that both CBD and CBG partially blocked the effect of JWH-133, which is a selective CB_2_R agonist. Similarly, both phytocannabinoids had a partial inhibitory effect on serotonin in 5HT1AR-expressing cells. When studying cells expressing CB_2_R–5HT_1A_R-Hets, it was observed that the impact of serotonin on recruiting β-arrestin 2-RLuc to the CB_2_R-YFP was marked, while the effect of selective CB_2_R agonist was negligible. In these cells, both CBD and CBG completely blocked the effect induced by serotonin. In HEK-293T cells expressing CB_2_R and 5HT_1A_R, the Gi-mediated signaling pathway was evaluated, showing that both JWH-133 and serotonin produced a substantial effect that was potentiated when administered together. Interestingly, CBD and CBG enhanced the effect of serotonin but not that of JWH-133 [62].

In the context of newborn hypoxic-ischemic brain damage, an increased expression of CB_2_R–5HT_1A_R-Hets has been reported in a pig model [71,72]. In order to investigate whether CBD treatment affects the expression levels of the CB_2_R–5HT_1A_R heteromer in an OGD environment, a proximity ligation assay (PLA) was conducted on striatal neurons. When the neurons were maintained in OGD conditions, striatal neurons exhibited a marked overexpression of CB_2_R–5HT_1A_R receptor complexes, proving that, in an episode of neuroinflammation, the heteromer is highly expressed. Notably, pretreatment with CBD and CBG led to a significant decrease in the expression of the receptor complex, indicating a potential neuroprotective effect of CBD [62].

PLA experiments were also conducted to study the expression of CB_2_R–5HT_1A_R receptor complexes on brain slices obtained from a rat hypoxic-ischemic model. The animals were subjected to carotid electrocoagulation and maintained in a hypoxic environment (10% O2) for 112 min and treated or not with CBD. Then, rats were sacrificed at 1, 7, or 30 days after the insult to assess the short- and long-term effects of the cannabinoid. Results showed that CBD treatment was able to reverse the upregulation of the receptor complex expression induced by hypoxia [62]. The expression of the receptor complex was markedly decreased in cerebral cortex sections taken 7 and 30 days after the lesion compared to the sections taken 1 day after the insult. Additionally, CBD administration resulted in a downregulation of heteroreceptor complex expression [62]. All together, these data indicate that CB_2_R–5HT_1A_R-Het expression is upregulated in OGD conditions and that phytocannabinoids, especially CBD, revert this effect.

## 5. Heteromeric Complexes in Stroke

Other GPCR heteromers that could have a role as therapeutic targets to address the neuroinflammation taking place in stroke are the complexes formed between cannabinoid CB_1_ and CB_2_ receptors and between adenosine A_2A_ and cannabinoid CB_2_ receptors. CB_1_–CB_2_ receptor heteromers (CB_1_R–CB_2_RHets) and A_2A_–CB_2_ receptor heteromers (A_2A_R–CB_2_RHets) have been shown to play a role in neurodegenerative diseases such as Alzheimer’s and Parkinson’s diseases, which are known to course with neuroinflammation [73,74].

In microglial cultures, a low expression of CB_2_R has been described, as opposed to CB_1_R, and a low expression of CB_1_R–CB_2_RHets was also found. However, when microglia were activated, both CB_2_R and CB_1_R–CB_2_RHets expression increased [74]. Signaling through CB_2_R in resting microglia in both Gi-dependent and independent pathways was almost negligible but, when microglia were activated with lipopolysaccharide (LPS) plus interferon gamma (IFNg) or with Aβ1-42 oligomers, CB_2_R-mediated signaling increased significantly. Similar results were observed in microglia obtained from the APP_Sw,Ind_ Alzheimer’s disease mouse model [75], that is, microglia from control animals showed results similar to those obtained from resting microglia, while cells from the transgenic animals showed increased CB_2_R-mediated signaling and an increase in CB_1_R–CB_2_RHets expression [74]. Expression of CB_1_R–CB_2_RHets was also explored in striatal sections from a Parkinson’s disease rat model [76], which showed an increase in the number of heteromers compared to control rats. In Parkinsonian rats that had also been treated with levo-DOPA and had developed dyskinesia, an even higher increase was found [74].

Altogether, these data suggest that the higher expression of CB_2_ receptors in activated microglial phenotypes could underlie the neuroprotective action of cannabinoids, as neuronal loss is virtually absent in transgenic models of Alzheimer’s disease. The significant increase in CB_1_R–CB_2_RHets expression in activated microglia poses these complexes as an attractive target with potential to regulate microglial polarization from the proinflammatory M1 to the neuroprotective M2 phenotype. In this sense, more efforts are needed to explore how cannabinoids could regulate the expression of M1 versus M2 markers.

A common feature of A_2A_ and CB_2_ receptors is that their expression is upregulated in microglia in Alzheimer’s disease patients [77,78].

Franco et al. described that A_2A_R and CB_2_R are capable of directly interacting, forming A_2A_R–CB_2_RHets [73]. They described how, due to this interaction, in resting microglia, A_2A_R activation blocks CB_2_R-mediated Gi signaling. When A_2A_R is blocked with a selective antagonist, the brake over CB_2_R is released and higher CB_2_R-mediated Gi signaling is observed. In activated microglia, this heteromer print was also detected, but, in accordance with the results reported above [74], CB_2_R-mediated signaling increased compared to resting microglia. In microglia obtained from the Alzheimer’s disease mouse model, the APP_Sw,Ind_ signaling outcome resembled that from activated microglia, while, in cells from control animals, similar results to those obtained in resting microglia were obtained [73]. When authors measured A_2A_R–CB_2_RHets expression, transgenic animals showed a marked increase in the number of heteromers compared to control animals [73]. 

In studies with activated microglia after stroke, CB_2_ is the receptor that has appeared as more important in regulating cell activation [32,54]. Thus, the upregulation of A_2A_–CB_2_Hets in activated microglia in principle seems detrimental, as the activation of A_2A_R blocks the beneficial effects of CB_2_R action. Neuroinflammatory responses that course with increases in adenosine, such as stroke, would lead to a decreased anti-inflammatory response, promoting neurodegeneration. A good approach to overcome this issue would be the use of A_2A_R antagonists, as it would avoid not only the action of adenosine on A_2A_Rs but also the block on CB_2_R signaling. In fact, an A_2A_R antagonist, istradefylline, is already being used to address the symptoms of Parkinson’s disease [79,80].

## 6. CBD Potential in Stroke

CBD is one of the most abundant extracts of the Cannabis sativa plant, in which it may represent up to 40% of cannabis extracts [81]. Studies suggest that the action of CBD is largely related to the human endocannabinoid system. According to the World Health Organization, CBD in its pure state does not appear to exhibit effects that indicate dependence potential, nor abuse [82]. To date, there is no evidence that cannabidiol reveals public health problems.

CBD is a negative allosteric modulator of cannabinoid receptors at the nanomolar range but, at high concentrations, CBD acts as a partial agonist [83,84]. However, CBD is able to interact with other elements of the endocannabinoid system, such as the enzyme fatty acid amide hydrolase (FAAH). CBD inhibits FAAH, increasing anandamide levels and enhancing the cannabinoid signal [85].

Surprisingly, Castillo et al. observed that CBD exhibited the capacity to mitigate necrotic and apoptotic injuries in forebrain slices obtained from neonatal mice exposed to OGD. The concurrent application of CBD with the CB_2_R antagonist AM-630 annulled all protective outcomes, implying the involvement of CB_2_R in the neuroprotective actions of CBD within the immature brain [86].

CBD has, in general, low activity in cannabinoid receptors and has been generally assumed to have a complex poly-pharmacological profile and to regulate the activity of different receptors and proteins. The phytocannabinoid can activate different molecular targets, acting (i) as an agonist of the serotonin 5-HT1A receptor [87], the TRPV1 receptor [88], and of the PPARy receptor [89]; (ii) as a partial agonist of dopamine D2-like receptors [90,91]; and (iii) as an antagonist of GPR55 [92]. CBD is also able to interact with µ opioid receptors (MOR) and δ opioid receptors (DOR), which are part of the opioid system and are closely related to pain [93,94]. CBD can also exert its effects through the purinergic system, as evidenced by Silva et al., who showed that CBD reduces NF-kB activity at concentrations closely linked to those inducing cell death. Conversely, the CBD analogue dimethyl-heptyl-cannabidiol (DMH-CBD) decreases NF-kB activity at nontoxic concentrations in an A_2A_R-dependent fashion [95]. In addition, the co-incubation of CBD with an A_2A_R antagonist, SCH58261, abolished all the protective effects of the phytocannabinoid in forebrain slices from newborn mice subjected to OGD. These data suggest that A_2A_R seems to be also involved in these neuroprotective effects of CBD [86].

**Figure 2:**
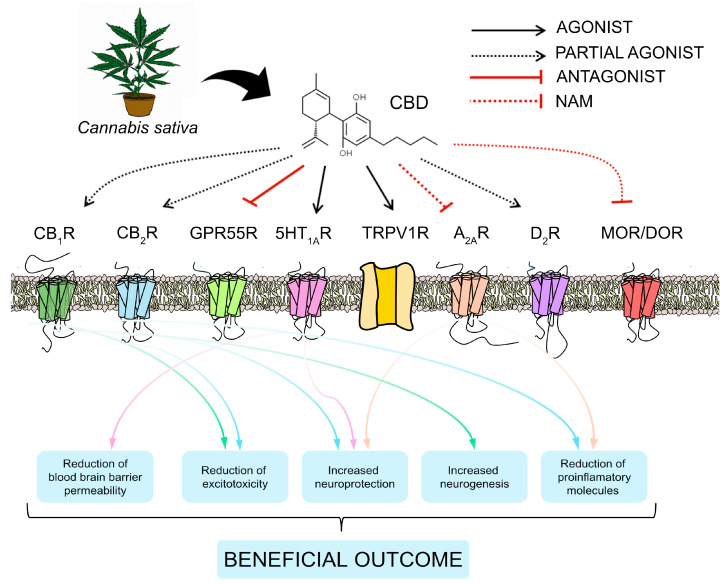
Beneficial effects of CBD through its action on different receptors. NAM: negative allosteric modulator.

Given its intricate pharmacology, CBD diverges from existing clinical therapeutic approaches by directly addressing the fundamental etiologies of vasogenic edema, notably the heightened permeability of the blood–brain barrier. CBD achieves this by diminishing blood–brain barrier permeability through activation of CB_1_, CB_2_, and 5-HT1A receptors. [96,97] and the neuroinflammation [98]. CBD modulates neuroinflammation through the reduction in proinflammatory molecules mediated by A_2_A and CB_2_ receptors [86], providing neuroprotection through CB_2_, A_2_A, and 5-HT1A receptors [99] and reducing excitoxicity through CB_1_ and CB_2_ receptors [97]. Furthermore, Wolf and collaborators highlighted the neurogenic effect of CBD through CB_1_R, adding to the beneficial effects of CBD in a therapeutic context [100] (Figure 2).

Preclinical studies have shown the effectiveness of CBD in mitigating the consequences of traumatic brain injury (TBI) and enhancing cerebral blood flow [101] and a reduction in genetic and pharmacologically induced seizures [102]. Several mechanisms can be involved in these effects, including an increase in cannabinoid signaling and a reduction in glutamate excitotoxicity [103], the promotion of neurogenesis [104], dampening of neuroinflammation [105], or scavenging reactive oxygen species [106].

Another critical consideration is whether CBD needs to have penetrated the brain before the injury occurs or if it might yield greater efficacy when administered during the response to the injury. Presently, conclusive evidence is lacking due to variations in CBD administration timing—some studies administer CBD prior to the primary lesion [103], others after [107], and yet others both before and after [96], with limited comparisons made between these different administration schedules. Despite this, in a gerbil model of ischemic stroke, the administration of CBD (1.25–20 mg/kg) 5 min after 10 min bilateral carotid occlusion allowed a complete survival of CA1 neurons (versus non-CBD animals), the 5 mg/kg dose showing the greatest neuroprotective effect [108]. The efficacy of CBD was investigated utilizing a middle cerebral artery occlusion (MCAO) model in neonatal rats. In this model, administration of CBD (3 mg/kg) following the insult decreased the volume of perilesional gliosis and reinstated long-term motor cognitive performance [109]. Also, pretreatment with CBD during five consecutive days before blocking the middle cerebral artery (MCA) during 60 min in male rats has antiapoptosis and antioxidant effects. The study outcomes demonstrated that CBD, administered at doses of 100 ng per rat, diminished the infarction volume and augmented the activity of endogenous antioxidant enzymes, such as superoxide dismutase and catalase, within the cerebral cortex and striatum [110].

It is true, however, that CBD acts on more than 65 receptors [111] and acts in the micromolar range on cannabinoid receptors. While CBD seems to be safe for both humans and animals [112,113], adverse effects have been documented when it was administered at relatively high doses (up to 50 mg kg^−1^ d^−1^). The prevailing occurrences (observed in >10% of patients receiving CBD treatment) included somnolence, diarrhea, diminished appetite, fatigue, pyrexia, vomiting, lethargy, upper respiratory tract infection, and convulsions [114,115,116].

Regarding the effects of CBD in astrocytes, in MCAO mice, a significant increase in intracellular Ca^2+^ in astrocytes associated with detrimental peri-infarct depolarizations has been observed. These intracellular Ca^2+^ oscillations in astrocytes occur in response to neuronal death and alarmin release and are detected in both the peri-infarct and penumbra zones [117]. CBD treatment can balance these ischemia-induced alterations in astroglial Ca^2+^ signaling after MCAO. In the same MCAO mouse model, 48 h post-ischemia, a much smaller area of astrocyte activation is detected compared to normal physiological conditions. However, in mice treated with CBD, the areas of these regions are much less affected [118]. Additionally, astrocytes change their morphology, becoming clasmatodendrocytes with shorter, thicker, and twisted branches. CBD treatment prevents astrocytes from undergoing these morphological changes [119]. Interestingly, CBD treatment (10 mg/kg, i.p.) decreases hippocampal reactivity of astrocytes and levels of GFAP 21 days after stroke in mice subjected to global cerebral ischemia [118].

Clinical investigation is imperative to ascertain the potential of CBD in mitigating or arresting the progression of symptoms precipitated by cerebral trauma and to evaluate its efficacy in shortening the convalescent period. However, preliminary evidence suggests that CBD holds promise in ameliorating ischemic stroke. Notably, the European Union has approved a clinical trial to test the use of CBD in the treatment of neonatal hypoxic-ischemic encephalopathy (neonatal HIE) (GWEP1560, EudraCT 2016-000936-17) [120], due to its capacity to augment the therapeutic efficacy of hypothermia in this condition [121,122,123].

## 7. Conclusions

Although it has been described that CB_1_R activation can show adverse effects, both cannabinoid receptors 1 and 2 have shown beneficial effects regarding the prognosis of ischemic stroke. Moreover, CB_2_R activation does not induce psychoactive effects; however, it has low expression levels. Phytocannabinoids not presenting psychoactive effects such as cannabidiol show an interesting potential to decrease neuroinflammation and neurodegeneration in animal models of hypoxia-ischemia, becoming a new promising therapy to improve stroke. New synthetic derivates of CBD should be evaluated with new approaches to try to find a compound showing the beneficial actions induced by cannabinoids without the non-desired side effects.

## Abbreviations

Δ^9^-THC	Δ^9^-tetrahydrocannabinol
2-AG	2-araquidonilglicerol
5HT_1A_R	serotonin receptor 1A
A_2A_R	adenosine receptor 2A
AEA	anandamide
BRET	bioluminescence resonance energy transfer
CB_1_R	cannabinoid receptor 1
CB_2_R	cannabinoid receptor 2
CBD	cannabidiol
CBG	cannabigerol
CNS	Central Nervous System
CVA	Cerebrovascular Accident
D_2_R	dopamine receptor 2
DMH-CBD	Dimethyl-Heptyl-Cannabidiol
DOR	δ opioid receptors
eMCAO	embolic middle cerebral artery occlusion
FAAH	fatty acid amide hydrolase
GPCR	G protein-coupled receptor
HIE	Neonatal Hypoxic-Ischemic Encephalopathy
IFNg	interferon gamma
LPS	lipopolysaccharide
MCA	middle cerebral artery
mTBI	mild traumatic brain injury
MOR	µ opioid receptors
OGD	oxygen and glucose deprivation
PLA	proximity ligation assay
pMCAO	permanent middle cerebral artery occlusion
PPARγ	peroxisome proliferator-activated receptor gamma
ROS	reactive oxygen species
TBI	traumatic brain injury
TRPV1	Transient Receptor Potential Vanilloid 1
ZO-1	tight junction protein zonula occludens-1

## References

[1] Warlow C.P. (1998). Epidemiology of Stroke. Lancet.

[2] Doyle K.P., Simon R.P., Stenzel-Poore M.P. (2008). Mechanisms of Ischemic Brain Damage. Neuropharmacology.

[3] Dhar R., Yu W., Yenari M., Moo J. (2023). Collateral Flow: Prolonging the Ischemic Penumbra. Transl. Stroke Res..

[4] Chavda V., Chaurasia B., Garg K., Deora H., Umana G.E., Palmisciano P., Scalia G., Lu B. (2022). Brain Disorders Molecular Mechanisms of Oxidative Stress in Stroke and Cancer. Brain Disord..

[5] Anthony S., Cabantan D., Monsour M., Borlongan C.V. (2022). Stroke Neuroinflammation, Stem Cells, and Stroke. Stroke.

[6] Qin C., Yang S., Chu Y., Zhang H., Pang X., Chen L., Zhou L., Chen M., Tian D., Wang W. (2022). Signaling Pathways Involved in Ischemic Stroke: Molecular Mechanisms and Therapeutic Interventions. Signal Transduct. Target. Ther..

[7] Béjot Y. (2022). Forty Years of Descriptive Epidemiology of Stroke. Neuroepidemiology.

[8] Rost N.S., Brodtmann A., Pase M.P., van Veluw S.J., Biffi A., Duering M., Hinman J.D., Dichgans M. (2022). Post-Stroke Cognitive Impairment and Dementia. Circ. Res..

[9] Chaves C.J., Caplan L.R. (2000). Heparin and Oral Anticoagulants in the Treatment of Brain Ischemia. J. Neurol. Sci..

[10] Lu D., Potter D.E. (2017). Cannabinoids and the Cannabinoid Receptors: An Overview. Handb. Cannabis Relat. Pathol. Biol. Pharmacol. Diagn. Treat..

[11] Caruso P., Naccarato M., Faoro V., Pracella D., Borando M., Dotti I., Koscica N., Stanta G., Pizzolato G., Manganotti P. (2016). Expression of the Endocannabinoid Receptor 1 in Human Stroke: An Autoptic Study. J. Stroke Cerebrovasc. Dis..

[12] Guo S., Liu Y., Ma R., Li J., Su B. (2016). Neuroprotective Effect of Endogenous Cannabinoids on Ischemic Brain Injury Induced by the Excess Microglia-Mediated Inflammation. Am. J. Transl. Res..

[13] Khaksar S., Bigdeli M.R. (2017). Correlation Between Cannabidiol-Induced Reduction of Infarct Volume and Inflammatory Factors Expression in Ischemic Stroke Model. Basic Clin. Neurosci..

[14] Fernández-Ruiz J., Moro M.A., Martínez-Orgado J. (2015). Cannabinoids in Neurodegenerative Disorders and Stroke/Brain Trauma: From Preclinical Models to Clinical Applications. Neurotherapeutics.

[15] Lai A.Y., Todd K.G. (2006). Microglia in Cerebral Ischemia: Molecular Actions and InteractionsThis Paper Is One of a Selection of Papers Published in This Special Issue, Entitled Young Investigator’s Forum. Can. J. Physiol. Pharmacol..

[16] Young A.P., Denovan-Wright E.M. (2022). The Dynamic Role of Microglia and the Endocannabinoid System in Neuroinflammation. Front. Pharmacol..

[17] Bernal-Chico A., Tepavcevic V., Manterola A., Utrilla C., Matute C., Mato S. (2023). Endocannabinoid Signaling in Brain Diseases: Emerging Relevance of Glial Cells. Glia.

[18] Mateo I., Pinedo A., Gomez-Beldarrain M., Basterretxea J.M., Garcia-Monco J.C. (2005). Recurrent Stroke Associated with Cannabis Use. J. Neurol. Neurosurg. Psychiatry.

[19] Tsatsakis A., Docea A.O., Calina D., Tsarouhas K., Zamfira L.M., Mitrut R., Sharifi-Rad J., Kovatsi L., Siokas V., Dardiotis E. (2019). A Mechanistic and Pathophysiological Approach for Stroke Associated with Drugs of Abuse. J. Clin. Med..

[20] Grambow E., Strüder D., Klar E., Hinz B., Vollmar B. (2016). Differential Effects of Endogenous, Phyto and Synthetic Cannabinoids on Thrombogenesis and Platelet Activity. BioFactors.

[21] Choi S.-H., Mou Y., Silva A.C. (2019). Cannabis and Cannabinoid Biology in Stroke. Stroke.

[22] Wolff V., Jouanjus E. (2017). Strokes Are Possible Complications of Cannabinoids Use. Epilepsy Behav..

[23] Amantea D., Spagnuolo P., Bari M., Fezza F., Mazzei C., Tassorelli C., Morrone L.A., Corasaniti M.T., Maccarrone M., Bagetta G. (2007). Modulation of the Endocannabinoid System by Focal Brain Ischemia in the Rat Is Involved in Neuroprotection Afforded by 17β-Estradiol. FEBS J..

[24] Muthian S., Rademacher D.J., Roelke C.T., Gross G.J., Hillard C.J. (2004). Anandamide Content Is Increased and CB1 Cannabinoid Receptor Blockade Is Protective during Transient, Focal Cerebral Ischemia. Neuroscience.

[25] Mechoulam R., Parker L.A. (2013). The Endocannabinoid System and the Brain. Annu. Rev. Psychol..

[26] Morales P., Goya P., Jagerovic N., Hernandez-Folgado L. (2016). Allosteric Modulators of the CB 1 Cannabinoid Receptor: A Structural Update Review. Cannabis Cannabinoid Res..

[27] Yang X., Wang X., Xu Z., Wu C., Zhou Y., Wang Y., Lin G., Li K., Wu M., Xia A. (2022). Molecular Mechanism of Allosteric Modulation for the Cannabinoid Receptor CB1. Nat. Chem. Biol..

[28] Kano M., Ohno-Shosaku T., Hashimotodani Y., Uchigashima M., Watanabe M. (2009). Endocannabinoid-Mediated Control of Synaptic Transmission. Physiol. Rev..

[29] Katona I., Freund T.F. (2012). Multiple Functions of Endocannabinoid Signaling in the Brain. Annu. Rev. Neurosci..

[30] Piomelli D. (2003). The Molecular Logic of Endocannabinoid Signalling. Nat. Rev. Neurosci..

[31] Caltana L., Saez T.M., Aronne M.P., Brusco A. (2015). Cannabinoid Receptor Type 1 Agonist ACEA Improves Motor Recovery and Protects Neurons in Ischemic Stroke in Mice. J. Neurochem..

[32] Schmidt W., Schäfer F., Striggow V., Fröhlich K., Striggow F. (2012). Cannabinoid Receptor Subtypes 1 and 2 Mediate Long-Lasting Neuroprotection and Improve Motor Behavior Deficits after Transient Focal Cerebral Ischemia. Neuroscience.

[33] Zhang R.L., Chopp M., Roberts C., Jia L., Wei M., Lu M., Wang X., Pourabdollah S., Zhang Z.G. (2011). Ascl1 Lineage Cells Contribute to Ischemia-Induced Neurogenesis and Oligodendrogenesis. J. Cereb. Blood Flow Metab..

[34] Hayakawa K., Mishima K., Nozako M., Hazekawa M., Aoyama Y., Ogata A., Harada K., Fujioka M., Abe K., Egashira N. (2007). High-Cholesterol Feeding Aggravates Cerebral Infarction via Decreasing the CB1 Receptor. Neurosci. Lett..

[35] Sommer C., Schomacher M., Berger C., Kuhnert K., Müller H.D., Schwab S., Schäbitz W.R. (2006). Neuroprotective Cannabinoid Receptor Antagonist SR141716A Prevents Downregulation of Excitotoxic NMDA Receptors in the Ischemic Penumbra. Acta Neuropathol..

[36] Reichenbach Z.W., Li H., Ward S.J., Tuma R.F. (2016). The CB1 Antagonist, SR141716A, Is Protective in Permanent Photothrombotic Cerebral Ischemia. Neurosci. Lett..

[37] Knowles M.D., de la Tremblaye P.B., Azogu I., Plamondon H. (2016). Endocannabinoid CB1 Receptor Activation upon Global Ischemia Adversely Impact Recovery of Reward and Stress Signaling Molecules, Neuronal Survival and Behavioral Impulsivity. Prog. Neuropsychopharmacol. Biol. Psychiatry.

[38] Parmentier-Batteur S., Jin K., Mao X.O., Xie L., Greenberg D.A. (2002). Increased Severity of Stroke in CB1 Cannabinoid Receptor Knock-out Mice. J. Neurosci..

[39] Youssef F.F., Hormuzdi S.G., Irving A.J., Frenguelli B.G. (2007). Cannabinoid Modulation of Neuronal Function after Oxygen/Glucose Deprivation in Area CA1 of the Rat Hippocampus. Neuropharmacology.

[40] Van Der Stelt M., Veldhuis W.B., Bär P.R., Veldink G.A., Vliegenthart J.F.G., Nicolay K. (2001). Neuroprotection by Delta9-Tetrahydrocannabinol, the Main Active Compound in Marijuana, against Ouabain-Induced in Vivo Excitotoxicity. J. Neurosci..

[41] Vendel E., de Lange E.C.M. (2014). Functions of the CB1 and CB2 Receptors in Neuroprotection at the Level of the Blood–Brain Barrier. NeuroMolecular Med..

[42] Eskla K.L., Vellama H., Tarve L., Eichelmann H., Jagomäe T., Porosk R., Oja V., Rämma H., Peet N., Laisk A. (2022). Hypothermia Alleviates Reductive Stress, a Root Cause of Ischemia Reperfusion Injury. Int. J. Mol. Sci..

[43] Hillard C. (2008). Role of Cannabinoids and Endocannabinoids in Cerebral Ischemia. Curr. Pharm. Des..

[44] Mendizábal V.E., Adler-Graschinsky E. (2007). Cannabinoids as Therapeutic Agents in Cardiovascular Disease: A Tale of Passions and Illusions. Br. J. Pharmacol..

[45] Patel S., Roelke C.T., Rademacher D.J., Cullinan W.E., Hillard C.J. (2004). Endocannabinoid Signaling Negatively Modulates Stress-Induced Activation of the Hypothalamic-Pituitary-Adrenal Axis. Endocrinology.

[46] Brennecke B., Gazzi T., Atz K., Fingerle J., Kuner P., Schindler T., Weck G.d., Nazaré M., Grether U. (2021). Cannabinoid Receptor Type 2 Ligands: An Analysis of Granted Patents since 2010. Pharm. Pat. Anal..

[47] Bie B., Wu J., Foss J.F., Naguib M. (2018). An Overview of the Cannabinoid Type 2 Receptor System and Its Therapeutic Potential. Curr. Opin. Anaesthesiol..

[48] Whiting Z.M., Yin J., de la Harpe S.M., Vernall A.J., Grimsey N.L. (2022). Developing the Cannabinoid Receptor 2 (CB2) Pharmacopoeia: Past, Present, and Future. Trends Pharmacol. Sci..

[49] Franco R., Morales P., Navarro G., Jagerovic N., Reyes-Resina I. (2022). The Binding Mode to Orthosteric Sites and/or Exosites Underlies the Therapeutic Potential of Drugs Targeting Cannabinoid CB2 Receptors. Front. Pharmacol..

[50] Lutz B. (2020). Neurobiology of Cannabinoid Receptor Signaling. Dialogues Clin. Neurosci..

[51] Tanaka M., Sackett S., Zhang Y. (2020). Endocannabinoid Modulation of Microglial Phenotypes in Neuropathology. Front. Neurol..

[52] Zou S., Kumar U. (2018). Cannabinoid Receptors and the Endocannabinoid System: Signaling and Function in the Central Nervous System. Int. J. Mol. Sci..

[53] Hashiesh H.M., Jha N.K., Sharma C., Gupta P.K., Jha S.K., Patil C.R., Goyal S.N., Ojha S.K. (2021). Pharmacological Potential of JWH133, a Cannabinoid Type 2 Receptor Agonist in Neurodegenerative, Neurodevelopmental and Neuropsychiatric Diseases. Eur. J. Pharmacol..

[54] Zarruk J.G., Fernández-López D., García-Yébenes I., García-Gutiérrez M.S., Vivancos J., Nombela F., Torres M., Burguete M.C., Manzanares J., Lizasoain I. (2012). Cannabinoid Type 2 Receptor Activation Downregulates Stroke-Induced Classic and Alternative Brain Macrophage/Microglial Activation Concomitant to Neuroprotection. Stroke.

[55] Bravo-Ferrer I., Cuartero M.I., Zarruk J.G., Pradillo J.M., Hurtado O., Romera V.G., Díaz-Alonso J., García-Segura J.M., Guzmán M., Lizasoain I. (2017). Cannabinoid Type-2 Receptor Drives Neurogenesis and Improves Functional Outcome after Stroke. Stroke.

[56] Yu S.-J., Reiner D., Shen H., Wu K.-J., Liu Q.-R., Wang Y. (2015). Time-Dependent Protection of CB2 Receptor Agonist in Stroke. PLoS ONE.

[57] Lakhan S.E., Kirchgessner A., Hofer M. (2009). Inflammatory Mechanisms in Ischemic Stroke: Therapeutic Approaches. J. Transl. Med..

[58] Cabañero D., Martín-García E., Maldonado R. (2021). The CB2 Cannabinoid Receptor as a Therapeutic Target in the Central Nervous System. Expert Opin. Ther. Targets.

[59] Turcotte C., Blanchet M.-R., Laviolette M., Flamand N. (2016). The CB2 Receptor and Its Role as a Regulator of Inflammation. Cell. Mol. Life Sci..

[60] Braun M., Khan Z.T., Khan M.B., Kumar M., Ward A., Achyut B.R., Arbab A.S., Hess D.C., Hoda M.N., Baban B. (2018). Selective Activation of Cannabinoid Receptor-2 Reduces Neuroinflammation after Traumatic Brain Injury via Alternative Macrophage Polarization. Brain. Behav. Immun..

[61] Murikinati S., Jüttler E., Keinert T., Ridder D.A., Muhammad S., Waibler Z., Ledent C., Zimmer A., Kalinke U., Schwaninger M. (2010). Activation of Cannabinoid 2 Receptors Protects against Cerebral Ischemia by Inhibiting Neutrophil Recruitment. FASEB J..

[62] Lillo J., Raïch I., Silva L., Zafra D.A., Lillo A., Ferreiro-Vera C., Sánchez de Medina V., Martínez-Orgado J., Franco R., Navarro G. (2022). Regulation of Expression of Cannabinoid CB2 and Serotonin 5HT1A Receptor Complexes by Cannabinoids in Animal Models of Hypoxia and in Oxygen/Glucose-Deprived Neurons. Int. J. Mol. Sci..

[63] Franco R., Villa M., Morales P., Reyes-Resina I., Gutiérrez-Rodríguez A., Jiménez J., Jagerovic N., Martínez-Orgado J., Navarro G. (2019). Increased Expression of Cannabinoid CB2 and Serotonin 5-HT1A Heteroreceptor Complexes in a Model of Newborn Hypoxic-Ischemic Brain Damage. Neuropharmacology.

[64] de Almeida D.L., Devi L.A. (2020). Diversity of Molecular Targets and Signaling Pathways for CBD. Pharmacol. Res. Perspect..

[65] Martínez-Pinilla E., Varani K., Reyes-Resina I., Angelats E., Vincenzi F., Ferreiro-Vera C., Oyarzabal J., Canela E.I., Lanciego J.L., Nadal X. (2017). Binding and Signaling Studies Disclose a Potential Allosteric Site for Cannabidiol in Cannabinoid CB2receptors. Front. Pharmacol..

[66] Navarro G., Reyes-Resina I., Rivas-Santisteban R., Sánchez de Medina V., Morales P., Casano S., Ferreiro-Vera C., Lillo A., Aguinaga D., Jagerovic N. (2018). Cannabidiol Skews Biased Agonism at Cannabinoid CB1 and CB2 Receptors with Smaller Effect in CB1-CB2 Heteroreceptor Complexes. Biochem. Pharmacol..

[67] Martínez-Aguirre C., Carmona-Cruz F., Velasco A.L., Velasco F., Aguado-Carrillo G., Cuéllar-Herrera M., Rocha L. (2020). Cannabidiol Acts at 5-HT1A Receptors in the Human Brain: Relevance for Treating Temporal Lobe Epilepsy. Front. Behav. Neurosci..

[68] England T.J., Hind W.H., Rasid N.A., O’Sullivan S.E. (2015). Cannabinoids in Experimental Stroke: A Systematic Review and Meta-Analysis. J. Cereb. Blood Flow Metab..

[69] Mishima K., Hayakawa K., Abe K., Ikeda T., Egashira N., Iwasaki K., Fujiwara M. (2005). Cannabidiol Prevents Cerebral Infarction Via a Serotonergic 5-Hydroxytryptamine 1A Receptor–Dependent Mechanism. Stroke.

[70] Kosari-Nasab M., Shokouhi G., Azarfarin M., Bannazadeh Amirkhiz M., Mesgari Abbasi M., Salari A.A. (2019). Serotonin 5-HT1A Receptors Modulate Depression-Related Symptoms Following Mild Traumatic Brain Injury in Male Adult Mice. Metab. Brain Dis..

[71] Lafuente H., Alvarez F.J., Pazos M.R., Alvarez A., Rey-Santano M.C., Mielgo V., Murgia-Esteve X., Hilario E., Martinez-Orgado J. (2011). Cannabidiol Reduces Brain Damage and Improves Functional Recovery After Acute Hypoxia-Ischemia in Newborn Pigs. Pediatr. Res..

[72] Pazos M.R., Mohammed N., Lafuente H., Santos M., Martínez-Pinilla E., Moreno E., Valdizan E., Romero J., Pazos A., Franco R. (2013). Mechanisms of Cannabidiol Neuroprotection in Hypoxic–Ischemic Newborn Pigs: Role of 5HT1A and CB2 Receptors. Neuropharmacology.

[73] Franco R., Reyes-Resina I., Aguinaga D., Lillo A., Jiménez J., Raïch I., Borroto-Escuela D.O., Ferreiro-Vera C., Canela E.I., de Medina V. (2019). Potentiation of Cannabinoid Signaling in Microglia by Adenosine A2A Receptor Antagonists. Glia.

[74] Navarro G., Borroto-Escuela D., Angelats E., Etayo Í., Reyes-Resina I., Pulido-Salgado M., Rodríguez-Pérez A.I., Canela E.I., Saura J., Lanciego J.L. (2018). Receptor-Heteromer Mediated Regulation of Endocannabinoid Signaling in Activated Microglia. Role of CB1 and CB2 Receptors and Relevance for Alzheimer’s Disease and Levodopa-Induced Dyskinesia. Brain. Behav. Immun..

[75] Mucke L., Masliah E., Yu G.Q., Mallory M., Rockenstein E.M., Tatsuno G., Hu K., Kholodenko D., Johnson-Wood K., McConlogue L. (2000). High-Level Neuronal Expression of Abeta 1-42 in Wild-Type Human Amyloid Protein Precursor Transgenic Mice: Synaptotoxicity without Plaque Formation. J. Neurosci. Off. J. Soc. Neurosci..

[76] Muñoz A., Garrido-Gil P., Dominguez-Meijide A., Labandeira-Garcia J.L. (2014). Angiotensin Type 1 Receptor Blockage Reduces L-Dopa-Induced Dyskinesia in the 6-OHDA Model of Parkinson’s Disease. Involvement of Vascular Endothelial Growth Factor and Interleukin-1β. Exp. Neurol..

[77] Angulo E., Casadó V., Mallol J., Canela E.I., Viñals F., Ferrer I., Lluis C., Franco R. (2003). A1 Adenosine Receptors Accumulate in Neurodegenerative Structures in Alzheimer Disease and Mediate Both Amyloid Precursor Protein Processing and Tau Phosphorylation and Translocation. Brain Pathol..

[78] Solas M., Francis P.T., Franco R., Ramirez M.J. (2013). CB2 Receptor and Amyloid Pathology in Frontal Cortex of Alzheimer’s Disease Patients. Neurobiol. Aging.

[79] Kondo T., Mizuno Y. (2015). Japanese Istradefylline Study Group A Long-Term Study of Istradefylline Safety and Efficacy in Patients with Parkinson Disease. Clin. Neuropharmacol..

[80] Mizuno Y., Kondo T. (2013). Japanese Istradefylline Study Group Adenosine A2A Receptor Antagonist Istradefylline Reduces Daily OFF Time in Parkinson’s Disease. Mov. Disord. Off. J. Mov. Disord. Soc..

[81] Rock E.M., Parker L.A. (2021). Constituents of Cannabis Sativa. Adv. Exp. Med. Biol..

[82] Expert Committee on Drug Dependence, WHO (2018). CANNABIDIOL (CBD) Critical Review Report.

[83] Franco R., Rivas-Santisteban R., Reyes-Resina I., Casanovas M., Pérez-Olives C., Ferreiro-Vera C., Navarro G., Sánchez de Medina V., Nadal X. (2020). Pharmacological Potential of Varinic-, Minor-, and Acidic Phytocannabinoids. Pharmacol. Res..

[84] Laprairie R.B., Bagher A.M., Kelly M.E.M., Denovan-Wright E.M. (2015). Cannabidiol Is a Negative Allosteric Modulator of the Cannabinoid CB1 Receptor. Br. J. Pharmacol..

[85] De Petrocellis L., Ligresti A., Moriello A.S., Allarà M., Bisogno T., Petrosino S., Stott C.G., Di Marzo V. (2011). Effects of Cannabinoids and Cannabinoid-Enriched Cannabis Extracts on TRP Channels and Endocannabinoid Metabolic Enzymes. Br. J. Pharmacol..

[86] Castillo A., Tolón M.R., Fernández-Ruiz J., Romero J., Martinez-Orgado J. (2009). The Neuroprotective Effect of Cannabidiol in an in Vitro Model of Newborn Hypoxic-Ischemic Brain Damage in Mice Is Mediated by CB2 and Adenosine Receptors. Neurobiol. Dis..

[87] Russo E.B., Burnett A., Hall B., Parker K.K. (2005). Agonistic Properties of Cannabidiol at 5-HT1a Receptors. Neurochem. Res..

[88] Bisogno T., Hanuš L., De Petrocellis L., Tchilibon S., Ponde D.E., Brandi I., Moriello A.S., Davis J.B., Mechoulam R., Di Marzo V. (2001). Molecular Targets for Cannabidiol and Its Synthetic Analogues: Effect on Vanilloid VR1 Receptors and on the Cellular Uptake and Enzymatic Hydrolysis of Anandamide. Br. J. Pharmacol..

[89] Jadoon K.A., Ratcliffe S.H., Barrett D.A., Thomas E.L., Stott C., Bell J.D., O’Sullivan S.E., Tan G.D. (2016). Efficacy and Safety of Cannabidiol and Tetrahydrocannabivarin on Glycemic and Lipid Parameters in Patients With Type 2 Diabetes: A Randomized, Double-Blind, Placebo-Controlled, Parallel Group Pilot Study. Diabetes Care.

[90] Seeman P. (2016). Cannabidiol Is a Partial Agonist at Dopamine D2High Receptors, Predicting Its Antipsychotic Clinical Dose. Transl. Psychiatry.

[91] Shrader S.H., Tong Y.G., Duff M.B., Freedman J.H., Song Z.H. (2020). Involvement of Dopamine Receptor in the Actions of Non-Psychoactive Phytocannabinoids. Biochem. Biophys. Res. Commun..

[92] Ross R.A. (2009). The Enigmatic Pharmacology of GPR55. Trends Pharmacol. Sci..

[93] Kathmann M., Flau K., Redmer A., Tränkle C., Schlicker E. (2006). Cannabidiol Is an Allosteric Modulator at Mu- and Delta-Opioid Receptors. Naunyn. Schmiedebergs. Arch. Pharmacol..

[94] Viudez-Martínez A., García-Gutiérrez M.S., Navarrón C.M., Morales-Calero M.I., Navarrete F., Torres-Suárez A.I., Manzanares J. (2018). Cannabidiol Reduces Ethanol Consumption, Motivation and Relapse in Mice. Addict. Biol..

[95] Silva R.L., Silveira G.T., Wanderlei C.W., Cecilio N.T., Maganin A.G.M., Franchin M., Marques L.M.M., Lopes N.P., Crippa J.A., Guimarães F.S. (2019). DMH-CBD, a Cannabidiol Analog with Reduced Cytotoxicity, Inhibits TNF Production by Targeting NF-KB Activity Dependent on A2A Receptor. Toxicol. Appl. Pharmacol..

[96] Jiang H., Li H., Cao Y., Zhang R., Zhou L., Zhou Y., Zeng X., Wu J., Wu D., Wu D. (2021). Effects of Cannabinoid (CBD) on Blood Brain Barrier Permeability after Brain Injury in Rats. Brain Res..

[97] Calapai F., Cardia L., Sorbara E.E., Navarra M., Gangemi S., Calapai G., Mannucci C. (2020). Cannabinoids, Blood–Brain Barrier, and Brain Disposition. Pharmaceutics.

[98] Dearborn J.T., Nelvagal H.R., Rensing N.R., Takahashi K., Hughes S.M., Wishart T.M., Cooper J.D., Wong M., Sands M.S. (2022). Effects of Chronic Cannabidiol in a Mouse Model of Naturally Occurring Neuroinflammation, Neurodegeneration, and Spontaneous Seizures. Sci. Rep..

[99] Campos A.C., Fogaça M.V., Sonego A.B., Guimarães F.S. (2016). Cannabidiol, Neuroprotection and Neuropsychiatric Disorders. Pharmacol. Res..

[100] Wolf S.A., Bick-Sander A., Fabel K., Leal-Galicia P., Tauber S., Ramirez-Rodriguez G., Müller A., Melnik A., Waltinger T.P., Ullrich O. (2010). Cannabinoid Receptor CB1 Mediates Baseline and Activity-Induced Survival of New Neurons in Adult Hippocampal Neurogenesis. Cell Commun. Signal..

[101] Crippa J.A.d.S., Zuardi A.W., Garrido G.E.J., Wichert-Ana L., Guarnieri R., Ferrari L., Azevedo-Marques P.M., Hallak J.E.C., McGuire P.K., Busatto G.F. (2004). Effects of Cannabidiol (CBD) on Regional Cerebral Blood Flow. Neuropsychopharmacology.

[102] Patra P.H., Barker-Haliski M., White H.S., Whalley B.J., Glyn S., Sandhu H., Jones N., Bazelot M., Williams C.M., McNeish A.J. (2019). Cannabidiol Reduces Seizures and Associated Behavioral Comorbidities in a Range of Animal Seizure and Epilepsy Models. Epilepsia.

[103] Santiago-Castañeda C., Huerta de la Cruz S., Martínez-Aguirre C., Orozco-Suárez S.A., Rocha L. (2022). Cannabidiol Reduces Short- and Long-Term High Glutamate Release after Severe Traumatic Brain Injury and Improves Functional Recovery. Pharmaceutics.

[104] Sales A.J., Fogaça M.V., Sartim A.G., Pereira V.S., Wegener G., Guimarães F.S., Joca S.R.L. (2019). Cannabidiol Induces Rapid and Sustained Antidepressant-Like Effects Through Increased BDNF Signaling and Synaptogenesis in the Prefrontal Cortex. Mol. Neurobiol..

[105] Yeisley D.J., Arabiyat A.S., Hahn M.S. (2021). Cannabidiol-Driven Alterations to Inflammatory Protein Landscape of Lipopolysaccharide-Activated Macrophages In Vitro May Be Mediated by Autophagy and Oxidative Stress. Cannabis Cannabinoid Res..

[106] Atalay S., Jarocka-Karpowicz I., Skrzydlewska E. (2019). Antioxidative and Anti-Inflammatory Properties of Cannabidiol. Antioxidants.

[107] Belardo C., Iannotta M., Boccella S., Rubino R.C., Ricciardi F., Infantino R., Pieretti G., Stella L., Paino S., Marabese I. (2019). Oral Cannabidiol Prevents Allodynia and Neurological Dysfunctions in a Mouse Model of Mild Traumatic Brain Injury. Front. Pharmacol..

[108] Braida D., Pegorini S., Arcidiacono M.V., Consalez G.G., Croci L., Sala M. (2003). Post-Ischemic Treatment with Cannabidiol Prevents Electroencephalographic Flattening, Hyperlocomotion and Neuronal Injury in Gerbils. Neurosci. Lett..

[109] Ceprián M., Jiménez-Sánchez L., Vargas C., Barata L., Hind W., Martínez-Orgado J. (2017). Cannabidiol Reduces Brain Damage and Improves Functional Recovery in a Neonatal Rat Model of Arterial Ischemic Stroke. Neuropharmacology.

[110] Khaksar S., Bigdeli M., Samiee A., Shirazi-zand Z. (2022). Antioxidant and Anti-Apoptotic Effects of Cannabidiol in Model of Ischemic Stroke in Rats. Brain Res. Bull..

[111] Guo Y., Wei R., Deng J., Guo W. (2024). Research Progress in the Management of Vascular Disease with Cannabidiol: A Review. J. Cardiothorac. Surg..

[112] Iffland K., Grotenhermen F. (2017). An Update on Safety and Side Effects of Cannabidiol: A Review of Clinical Data and Relevant Animal Studies. Cannabis Cannabinoid Res..

[113] Machado Bergamaschi M., Helena Costa Queiroz R., Waldo Zuardi A., Alexandre S., Crippa J. (2011). Safety and Side Effects of Cannabidiol, a Cannabis Sativa Constituent. Curr. Drug Saf..

[114] Devinsky O., Marsh E., Friedman D., Thiele E., Laux L., Sullivan J., Miller I., Flamini R., Wilfong A., Filloux F. (2016). Cannabidiol in Patients with Treatment-Resistant Epilepsy: An Open-Label Interventional Trial. Lancet Neurol..

[115] O’Connell B.K., Gloss D., Devinsky O. (2017). Cannabinoids in Treatment-Resistant Epilepsy: A Review. Epilepsy Behav..

[116] Tzadok M., Uliel-Siboni S., Linder I., Kramer U., Epstein O., Menascu S., Nissenkorn A., Yosef O.B., Hyman E., Granot D. (2016). CBD-Enriched Medical Cannabis for Intractable Pediatric Epilepsy. Seizure.

[117] Rakers C., Petzold G.C. (2016). Astrocytic Calcium Release Mediates Peri-Infarct Depolarizations in a Rodent Stroke Model. J. Clin. Investig..

[118] Meyer E., Rieder P., Gobbo D., Candido G., Scheller A., de Oliveira R.M.W., Kirchhoff F. (2022). Cannabidiol Exerts a Neuroprotective and Glia-Balancing Effect in the Subacute Phase of Stroke. Int. J. Mol. Sci..

[119] Lana D., Landucci E., Mazzantini C., Magni G., Pellegrini-Giampietro D.E., Giovannini M.G. (2022). The Protective Effect of CBD in a Model of In Vitro Ischemia May Be Mediated by Agonism on TRPV2 Channel and Microglia Activation. Int. J. Mol. Sci..

[120] Martínez-Orgado J., Villa M., del Pozo A. (2021). Cannabidiol for the Treatment of Neonatal Hypoxic-Ischemic Brain Injury. Front. Pharmacol..

[121] Barata L., Arruza L., Rodríguez M.-J., Aleo E., Vierge E., Criado E., Sobrino E., Vargas C., Ceprián M., Gutiérrez-Rodríguez A. (2019). Neuroprotection by Cannabidiol and Hypothermia in a Piglet Model of Newborn Hypoxic-Ischemic Brain Damage. Neuropharmacology.

[122] Burstein S. (2015). Cannabidiol (CBD) and Its Analogs: A Review of Their Effects on Inflammation. Bioorg. Med. Chem..

[123] Lafuente H., Pazos M.R., Alvarez A., Mohammed N., Santos M., Arizti M., Alvarez F.J., Martinez-Orgado J.A. (2016). Effects of Cannabidiol and Hypothermia on Short-Term Brain Damage in New-Born Piglets after Acute Hypoxia-Ischemia. Front. Neurosci..

